# Comparative Transcriptomic Analysis of Rectal Tissue from Beef Steers Revealed Reduced Host Immunity in *Escherichia coli* O157:H7 Super-Shedders

**DOI:** 10.1371/journal.pone.0151284

**Published:** 2016-03-09

**Authors:** Ou Wang, Guanxiang Liang, Tim A. McAllister, Graham Plastow, Kim Stanford, Brent Selinger, Le Luo Guan

**Affiliations:** 1 Department of Agricultural, Food and Nutritional Science, University of Alberta, Edmonton, AB, Canada; 2 Agriculture and Agri-Food Canada, Lethbridge Research Centre, Lethbridge, AB, Canada; 3 Alberta Agriculture and Forestry, Lethbridge, AB, Canada; 4 Biological Sciences Department, University of Lethbridge, Lethbridge, AB, Canada; University of Florida, UNITED STATES

## Abstract

Super-shedder cattle are a major disseminator of *E*. *coli* O157:H7 into the environment, and the terminal rectum has been proposed as the primary *E*. *coli* O157:H7 colonization site. This study aimed to identify host factors that are associated with the super-shedding process by comparing transcriptomic profiles in rectal tissue collected from 5 super-shedder cattle and 4 non-shedder cattle using RNA-Seq. In total, 17,859 ± 354 genes and 399 ± 16 miRNAs were detected, and 11,773 genes were expressed in all animals. Fifty-eight differentially expressed (DE) genes (false discovery rate < 0.05) including 11 up-regulated and 47 down-regulated (log 2 (fold change) ranged from -5.5 to 4.2), and 2 up-regulated DE miRNAs (log 2 (fold change) = 2.1 and 2.5, respectively) were identified in super-shedders compared to non-shedders. Functional analysis of DE genes revealed that 31 down-regulated genes were potentially associated with reduced innate and adaptive immune functions in super-shedders, including 13 lymphocytes membrane receptors, 3 transcription factors and 5 cytokines, suggesting the decreased key host immune functions in the rectal tissue of super-shedders, including decreased quantity and migration of immune cells such as lymphocytes, neutrophils and dendritic cells. The up-regulation of bta-miR-29d-3p and the down regulation of its predicted target gene, *regulator of G-protein signaling 13*, suggested a potential regulatory role of this miRNA in decreased migration of lymphocytes in super-shedders. Based on these findings, the rectal tissue of super-shedders may inherently exhibit less effective innate and adaptive immune protection. Further study is required to confirm if such effect on host immunity is due to the nature of the host itself or due to actions mediated by *E*. *coli* O157:H7.

## Introduction

*Escherichia coli* O157:H7 is a foodborne pathogen that can cause disease in humans, including abdominal cramps, bloody diarrhea and hemolytic uremic syndrome. It is estimated that this bacterium causes more than 60,000 illnesses in the U.S. annually [1]. Cattle are the main reservoir of *E*. *coli* O157:H7, and many *E*. *coli* O157:H7 outbreaks have been traced to contaminated meat, fruits or vegetables that have direct contact with cattle feces or contaminated water [2]. Cattle shedding more than 10^4^ CFU *E*. *coli* O157:H7 per gram of feces are defined as “super-shedders” (SS) [3]. It is speculated that these individuals are the source of the majority of *E*. *coli* O157:H7 cells that enter the environment and/or food processing chain [4]. Three types of super-shedders have been described, which are non-persistent shedders (shedding lasts ~14 days after *E*. *coli* O157:H7 challenge), moderately persistent shedders (lasts ~30 days), and persistent shedders (lasts several months) [5]. Many studies have reported that the recto-anal junction (RAJ) of cattle is the primary colonization site of *E*. *coli* O157:H7, with the formation of biofilms at this location playing an integral role in super-shedding [6]. Although the mechanism of the super-shedding phenomenon is large unknown, many factors such as diet, seasonality, environment, cattle host, and interactions between the microbiome and the intestinal epithelium as well as interactions within the microbiome have been considered to affect this phenomenon [6].

The interactions between *E*. *coli* O157:H7 and the immune system of cattle have been previously reviewed [7]. In general, the flagellum of *E*. *coli* O157:H7 has been suggested to initiate the colonization of the bovine epithelium, and upon colonization the type III secretion system injects effector proteins, including intimin receptor (Tir), *Escherichia* secretion protein (EspA, EspB and EspD) into epithelial cells. In response, the bovine intestinal epithelial cells recognize the *E*. *coli* O157:H7 flagellum with toll-like receptor 5 (TLR5) activating NF-κB pathways. Lipopolysaccharide (LPS) from *E*. *coli* O157:H7 can also be recognized by macrophages and bovine colonic cells, based on the evidence on altered gene expression in cultured cells. Adaptive immune protection can be mounted in cattle against *E*. *coli* O157:H7, including IgG and IgA antibodies against Stx1 (Shiga-like toxin), Stx2, LPS and intimin, as well as *E*. *coli* O157:H7 secretive proteins. Previous gene expression measurement for rectal tissue of cattle shedding *E*. *coli* O157 indicated that the changes in gene expression related to immune responses may be sustained while the number of fecal *E*. *coli* O157 decreases, although the maximum changes in expression may occur when the number of *E*. *coli* O157 peaks [8]. Interestingly, a study challenging calves and peripheral blood mononuclear cells with *E*. *coli* O157 revealed that the Stx produced by *E*. *coli* O157:H7 is able to inhibit the proliferation of lymphocytes [9].

To date, little studies have examined the contributions of host mechanisms at the RAJ to the growth of *E*. *coli* O157:H7 and the super-shedding phenomenon. Antibody secretion and immune cell infiltration and changes in gene expression at this site have been observed in cattle challenged with *E*. *coli* O157:H7 [7]. However, such changes at the RAJ have not been examined in cattle that are natural super-shedders. In this study, we hypothesized that the gene/miRNA expression profiles of RAJ tissue differ between SS and non-shedders (NS) cattle. Comparative transcriptome analysis of RAJ tissues collected from cattle previously shown to be SS and NS [10] was performed using high throughput RNA-Seq.

## Materials and Methods

The animal study was approved by the Animal Care Committee of Lethbridge Research Centre, Agriculture Agri-food Canada (Animal Care Committee protocol number: 1120).

### Identification of SS cattle and rectum tissue collection

Fecal samples were collected from 400 British x Continental feedlot steers (452 kg ± 23 kg) that were fed a barley-grain based finishing diet and cared for in accordance with the guidelines of the Canadian Council of Animal Care (Animal Care Committee protocol number: 1120) [10]. A fecal sample (50 g) from each steer was collected from the rectum, transferred to a sterile container, placed on ice and immediately transported to the laboratory for enumeration using culture-based methods as outlined previously [10]. Briefly, individually collected samples were plated for microbial analysis within 4 h after sample collection. The fecal sample (1 g) was serial diluted with 9 mL of phosphate buffered saline (PBS) from 10^−1^ to 10^−4^. The dilutions were plated onto sorbitol MacConkey agar with 2.5 mg/L potassium tellurite and 0.05 mg/L cefixime (CT-SMAC; Dalynn Biologicals, Calgary, AB), and incubated at 37°C for 18 to 24 h. A colony counter (Reichert, Depew, NY) was used for bacterial enumeration (colony forming unit, CFU), and steers identified with ≥10^4^ CFU of *E*. *coli* O157:H7 per gram of feces were defined as SS. The CT-SMAC selected isolates were then examined using an *E*. *coli* O157 Latex Test kit (Oxoid Ltd, Basingstoke, Hampshire, UK) following the manufacturer’s instruction, and the positive agglutination isolates were further verified using a multiplex PCR assay targeting genes encoding virulent factors including verotoxin (*vt*), intimine (*eaeA)*, H7 flagellin (*fliC*)) as described previously [11] to define the *E*. *coli* O157:H7 serotype. Based on above evaluation, 5 SS and 4 control pen-mates which tested negative for *E*. *coli* O157:H7 (NS) were selected for slaughter. Prior slaughter, the fecal samples of SS were collected twice daily for 4–10 days, and the fecal *E*. *coli* O157:H7 was monitored using the cultured based method as described above and subsequently an immunomagnetic separation assay using anti-*E*. *coli* O157 Dynabeads (Invitrogen, Carlsbad, CA) following manufacturer's instructions as outlined in a previous study [10]. When animals were slaughtered, two 2 cm^2^ biopsies 3–5 cm proximal to the RAJ were collected and immediately flash frozen in liquid nitrogen within 10 min after death of the animal. Samples were stored at -80°C until RNA was extracted.

### RNA extraction

Before RNA extraction, frozen RAJ tissue samples from 5 SS and 4 NS were ground to a fine powder in liquid nitrogen using a pestle and mortar. Total RNA was extracted from about 100 mg of powdered tissue using a mirVana total RNA Isolation Kit (Ambion, Carlsbad, CA, USA) following the manufacturer’s instructions. The Agilent 2200 TapeStation (Agilent Technologies, Santa Clara, CA, USA) and Qubit 2.0 Fluorometer (Invitrogen, Carlsbad, CA, USA) were used to measure RNA quality and quantity respectively. RNA with an integrity number (RIN) greater than 7.0 (and ratio of 28S/18S ranging from 1.7 to 2.4) was used for RNA-Seq library construction.

### RNA-Seq/miRNA library preparation and sequencing

Extracted total RNA (1 μg) was used for library preparation using a Truseq Stranded Total RNA Sample Preparation kit (Illumina, San Diego, CA, USA) following the manufacturer’s instructions. Firstly, the rRNAs were depleted using biotinylated, target-specific oligos combined rRNA removal beads, and the remaining RNA was fragmented followed by first strand cDNA synthesis with reverse transcriptase and random primers. For the second strand cDNA synthesis, DNA polymerase I and RNase H were used, followed by ligation of the indexed-adapters and PCR enrichment (98°C for 30 sec, followed by 15 cycles of: 98°C for 10 sec, 60°C for 30 sec, 72°C for 30 sec, and 72°C for 5 min; the final products were held at 4°C). To ensure that the size of the products was around 250–270 bp and the concentration was adequate for sequencing, libraries were measured using a Agilent 2200 TapeStation and a Qubit 2.0 Fluorometer, respectively. In addition, miRNA libraries were constructed using a TruSeq Small RNA sample preparation kit (Illumina, San Diego, CA) and 1.0 μg of extracted total RNA was used for library preparation. RNA sequencing was performed using a HiSeq 2000 sequencing system (Illumina, San Diego, CA, USA), with paired-end (100 bp) sequencing for mRNA and single read (50 bp) for miRNA at Genome Quebec Innovation Centre, Montreal, Quebec, Canada.

### mRNA profiling and differential expression analysis

Sequencing reads were first mapped against the reference bovine genome UMD3.1 (DNA source was Hereford cattle) assembly [12] using Tophat2 [13]. The Tophat2 splice aligner was used to address the two most challenging issues in the mapping of RNA-Seq reads: (1) reads spanning multiple exons, and (2) reads more readily mapping to pseudogenes than to their real positions [13]. To study gene expression profiles of RAJ tissues, the read counts mapped to the genome were normalized into FPKM (fragments per kilobase of exon per million fragments mapped) using Cufflinks [14].

The Cufflinks pipeline [14] was used for differentially expressed (DE) gene identification. Cufflinks takes the raw-reads alignment output from Tophat2 and assembles the mapped reads into transcripts to construct a transcriptome for each sample. The reads were assembled into transcripts by Cufflinks, and the transcripts were annotated using Ensembl umd3.1.GTF file (ftp://ftp.ensembl.org/pub/release-80/gtf/bos_taurus). After transcriptome assembly, Cuffmerge was used to merge the transcriptomes into a uniform basis for expression level calculation. The DE analysis were then performed using Cuffdiff with FPKM ≥ 0.3, FDR < 0.05 and log 2 (fold change) > 1 or < -1 as cut-off, which have been applied in other RNA-Seq based studies [15,16]. Only the DE genes expressed in at least 50% of NS and SS, were retained and subjected to functional analysis.

### miRNA profiling and differential expression analysis

miRNA sequencing data were analyzed using sRNA toolbox which includes several independent but interconnected tools for miRNA expression (based on sequences from miRBase [17]), differential expression analysis and target gene prediction [18]. The read counts of identified miRNAs were normalized into reads per million (RPM). Bioconductor package edgeR [19], which provides statistical methods for identification of differentially expressed genes/miRNAs was used for DE miRNA identification, and log 2 (fold change) > 1 or < -1 and FDR < 0.1 were used as cut-offs [20].

Function analysis of DE miRNAs were performed by prediction of their targets using PITA (Probability of Interaction by Target Accessibility) [21] and miRanda [22] packages. Pearson correlation coefficients were calculated between the expression values of miRNA and their target genes. Only the miRNA-target pairs that showing a negative correlation (r < -0.6, P-value < 0.05) were retained for downstream analysis.

### Functional analysis of rectal transcriptome and differentially expressed genes

Functional analysis of the transcriptome of RAJ tissue and DE genes were performed using Ingenuity Pathway Analysis® (IPA, IPA®, QIAGEN Redwood City, www.qiagen.com/ingenuity) with the Ingenuity Knowledge Base (gene only) used as a reference set for our analysis. The function of the core transcriptome of RAJ tissue was based on the top 8,000 genes with the greatest FPKM values (normalized abundance) as IPA does not support analyses with more than 8000 molecules. This software package performs a downstream effect analysis for the biological influence of the input gene set (the transcriptome or DE genes), which first identifies biological function, and then predicts if it is increased or decreased based on up- or down-regulation of genes. A z-score generated from IPA analysis is used to indicate the direction of change of a certain function, with z-scores ≥ 2.0 or ≤ -2.0 indicating the significant increase or decrease in a function, respectively. DAVID Bioinformatics [23] and KEGG_PATHWAY [24] were used for enrichment of biological pathways, and pathway analysis, respectively.

### Validation of gene expression using quantitative real time PCR (qPCR)

The main purpose to perform qPCR on gene expression is to validate the reliability of differential expression identified from RNA-Seq data. Ten identified DE genes related to immune function from RNA-Seq analysis were selected for qPCR validation, including chemokine (*C-C motif*) *ligand 21* (*CCL21*), *chemokine* (*C-C motif*) *receptor 7* (*CCR7*), *CD22 molecule* (*CD22*), *chemokine* (*C-X-C motif*) *ligand 13* (*CXCL13*), *lymphotoxin beta* (*LTB*), *CD19 molecule* (*CD19*), *interleukin 2 receptor*, *alpha* (*IL2RA*), *SH2 domain protein 1A* (*SH2D1A*), *POU class 2 associating factor 1 (POU2AF1)* and *4-domains*, *subfamily A*, *member 1* (*MS4A1*). The primers (Table 1) were designed using NCBI primer blast, and the primer pair specificity was verified by sequencing the PCR products (Table 1) before qPCR analysis.

**Table 1:** Primer sequences, amplicon sizes and annealing temperatures for qPCR assays.

Genes	Oligo sequence (5' to 3')	Amplicon size, bp	Access. No.**	Annealing temp,°C
***CCL21***	F: GCTATCCTGTTCTCGCCTCG	222	NM_001038076.2	60
	R: ACTGGGCTATGGCCCTTTTG			
***CCR7***	F: ACCCTCGCTAGCTACCTCAA	293	NM_001024930.3	64
	R: CGGTCTCTTGTCTTGGGGAC			
***CD22***	F: ACCTCAGTTTCCAGCCCAAG	188	XM_003587236.2	64
	R: CCTCATGGTCACAGACTCGC			
***CXCL13***	F: AACCCTCAAGCCAAATGGACA	154	NM_001015576.2	60
	R: AACCCGGAGCAGGAATGTTG			
***LTB***	F: TGGGAAGAGGAGGTCAGTCC	215	XM_002697371.2	62
	R: TAGCTTGCCATAAGTCGGGC			
***MS4A1***	F: GCGGAGAAGAACTCCACACA	206	NM_001077854.2	64
	R: GGGTTAGCTCGCTCACAGTT			
***IL2RA***	F: GCACGGTCAGGCTTCAGAT	288	NM_174358.2	64
	R: TTCTTGACTTCTTCTGGCCTTG			
***CD19***	F: CTCCCATACCTCCCTGGTCA	127	NM_001245998.1	64
	R: GCCCATGACCCACATCTCTC			
***POU2AF1***	GAGACCATGGTGACTGGTGG	246	NM_001075915.1	62
	AATACGGCCATTGTGGGGAG			
***SH2D1A***	CAGCACCGGGGGTACATAAA	146	NM_001034733.2	62
	TCCTGTAGCACCTTGTGTACTT			
β-actin*	F: CTAGGCACCAGGGCGTAATG	177	AF191490.1	60
	R: CCACACGGAGCTCGTTGTAG			

* Primer sequence from [69]

**NCBI accession number.

Total RNA (1 μg) was first reverse transcribed to single stranded cDNA using Oligo (dT)_12-18_ (Life Technologies, Carlsbad, CA, USA) and SuperScript^TM^ II RT (Life Technologies, Carlsbad, CA, USA). Quantitative real time PCR was performed using a StepOnePlus™ Real-Time PCR System (Applied Biosystems, Foster City, CA, USA) using SYBR® Green (Life Technologies, Carlsbad, CA, USA) chemistry and the following program: 95°C for 20 sec, followed by 40 cycles of denaturation at 95°C for 3 sec, and annealing/extension for 30 sec. The annealing temperature varied for each gene (Table 1). Four commonly used reference genes, bovine *GAPDH*, *18S rRNA*, *RPLP0* and *β-actin* [25], were used for qPCR analysis. Of the genes examined, *β-actin* had the most consistent Cq across all samples, and therefore it was used as the standard reference gene for comparison purposes. ΔCq value was used to indicate the relative quantity of target genes, which was calculated with the equation: ΔCq = Cq_target genes_-Cq_referece gene_. Statistical analysis was performed using two-sample t-test with SAS (version 9.2; SAS Institute Inc., Cary, NC) to compare the ΔCq of NS and SS.

### Availability of sequencing data

The RNA-Seq and miRNA-Seq data are available at NCBI Gene Expression Omnibus (GEO) database under accession number GSE67099 and GSE74627, respectively.

## Results and Discussion

### Identification of super-shedders

Eleven SS were identified from 400 animals (2.75% of sampled cattle) and the 5 individuals that exhibited the highest shedding levels were slaughtered (Table 2). Although these five individuals were not all shedding > 10^4^ CFU/g feces at the time of slaughter, they were confirmed as SS within the previous ten days. Cattle in the present study meet the definition of a non-persistent super-shedder as outlined previously [5]. In addition, although the tissue sampling was performed after SS identification and the number of fecal *E*. *coli* O157 decreased afterwards in the SS, it does not undermine the validity of using collected tissue samples for super-shedding phenomenon investigation. The changes in gene expression potentially induced by *E*. *coli* O157 can last even after the *E*. *coli* O157 shedding drops under super-shedding level [8]. The tissue sampling was managed to be accomplished after cattle were purchased from the commercial feedlot, within 4–10 days after the animals were identified as super-shedders, and all the super-shedders were at least positive for *E*. *coli* O157 before sampling. Therefore, the detected changes in gene expression may be still potentially related to *E*. *coli* O157 colonization.

**Table 2:** *Escherichia coli* O157:H7 numbers (log_10_ CFU/g feces)*.

Steer ID	*E*. *coli* O157:H7 numbers at the day of sampling, log CFU/g feces	Length of time between the first sampling and the day of slaughter, days	*E*. *coli* O157:H7 numbers day of/prior to slaughter, log CFU/g feces
**274_SS**	6.7	8	+** (a day prior to slaughter)
**287_SS**	5.4	8	+ (a day prior to slaughter)
**294_SS**	5.8	10	+ (a day prior to slaughter)
**299_SS**	7.8	10	+ (a day prior to slaughter)
**310_SS**	7.5	4	5.8 (day of slaughter)

*E. *coli* O157:H7 number in fecal samples of steers identified as super-shedders at the day of sampling and day of/prior to slaughter.

**+, positive for *E*. *coli* O157:H7 via immunomagnetic separation assay.

### Transcriptome profiling of bovine rectal tissue

The whole tissue of the RAJ was used for transcriptome profiling using RNA-Seq as the lymphoid structures underlying epithelial tissues might also influence super-shedding [26]. In total, the number of paired-end reads generated from parallel sequencing of RAJ tissues ranged from 25.5 M to 38.2 M (Table 3), with 17,859 ± 354 genes identified (at least one read mapped to the gene in at least one animal). The core transcriptome of RAJ tissue included 11,773 genes (with FPKM ≥ 0.03 in all animals).

**Table 3:** Sequencing results and percentage of reads mapped to reference genome by Tophat2.

Library name (Steer ID)	No. of Reads generated	Average Quality score	No. of reads mapped to reference	% of mapped reads
**108_NS**	38.2M	35	30.3M	79.2
**152_NS**	25.6M	35	20.1M	78.5
**165_NS**	27.6M	35	22.9M	82.9
**242_NS**	30.1M	35	23.5M	78.2
**274_SS**	27.0M	35	16.5M	61.1
**287_SS**	28.3M	35	24.1M	85.3
**294_SS**	28.7M	36	21.7M	75.5
**299_SS**	27.2M	36	21.0M	77.2
**310_SS**	35.0M	35	27.7M	79.2

**Fig 1:**
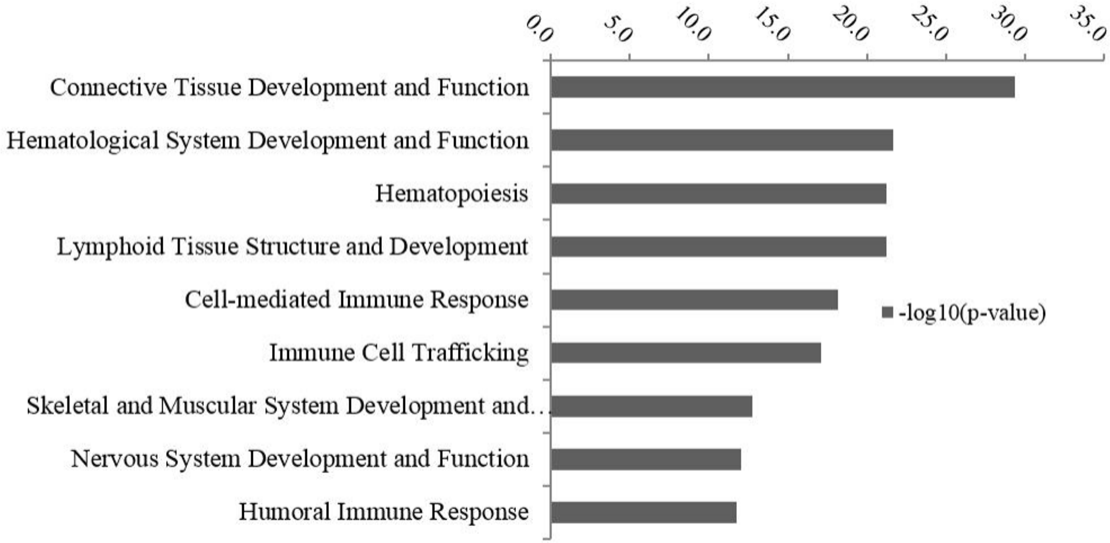
Top enriched functional terms (physiological process) by IPA for the core transcriptome of rectal anal junction (RAJ). The y-axis is on–log_10_ (P-value) scale: the greater the–log_10_ (P-value), the more likely that function is associated with the core transcriptome of the RAJ.

The top physiological functions associated with the core transcriptome (analyzed from top abundant 8,000 genes in S1 Table) of RAJ tissue is shown in Fig 1. A summary of the genes involved in each functional category is included in S2 Table and the identified function was plotted in -log_10_ (p-value) scale, with the p-value measuring if the association between a set of uploaded genes and a given function was due to random chance (Fig 1). The identified basic physiological functions fit this tissue since the rectum contains muscles, blood vessels (arteries, veins), lymphatics, and nerve fibers [27]. The main physiological function of the RAJ is to store and eliminate feces through peristalsis under the control of the nervous system [28]. As shown in Fig 1, genes associated with the development of the muscular (involved 275 genes) and nervous system (involved 562 genes) are included the core transcriptome of the rectal tissue. Considering that connective tissue is one of main components of the mucosa, submucosa, muscularis and adventitia of the RAJ, it is not surprising that “connective tissue development and function” was one of the top physiological processes (Fig 1). Other identified prominent physiological functions included hematological system development (1,145 genes), lymphoid tissue structure development (526 genes), and immune cell trafficking (580 genes). These observations correspond to the highly vascularized nature [27] of the RAJ and the presence of lymphoid follicles (ILFs) and lamina propria lymphocytes at this site as identified in cattle [26].

### Differentially expressed genes in RAJ between super-shedders and non-shedders and qPCR validation

**Fig 2:**
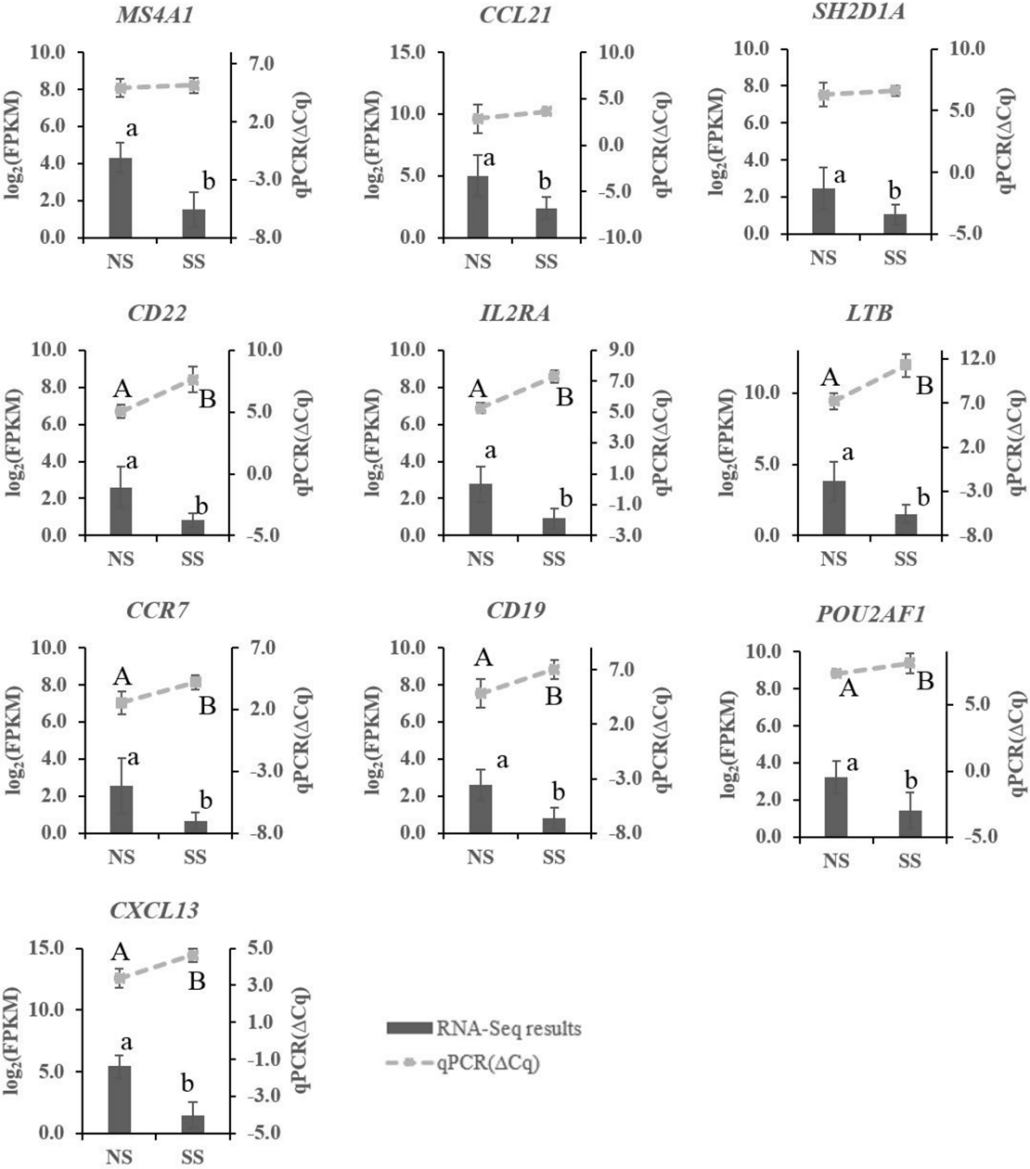
Expression of selected differentially expressed (DE) genes as detected by qRT-PCR and RNA-Sequencing. Differentially expressed genes as measured by qPCR are shown by lines on the top and values are indicated by the right Y-axis as relative expression level (ΔCq). Lower ΔCt values represent higher gene expression levels and *vice versa*. Gene expression as measured by RNA-Seq are shown by bar graphs on the bottom and values are indicated by the left Y-axis as log2 (FPKM). A, B indicate a significant difference in the relative expression detected by qPCR (P-value < 0.05); a, b indicate a significant difference in expression of genes detected by RNA-Seq (FDR < 0.05). Data are presented as mean ± standard deviation. NS and SS represent non-shedders and super-shedders, respectively.

Based on DE analysis, a total of 58 DE genes were identified between SS and NS, with 11 up-regulated and 47 down-regulated genes in SS, with log 2 (fold change) ranging from -5.5 to 4.2 (S3 Table). Among ten genes selected for qPCR, seven of them including *CCR7*, *CD22*, *CXCL13*, *LTB*, *IL2RA*, *CD19*, and *SH2D1A* showed significantly lower expression in SS comparing to NS, similar as identified by RNA-Seq (Fig 2). While expressions of *MS4A1*, *POU2AF1* and *CCL21* were not significantly different between SS and NS, their ΔCq in SS was greater than in NS, indicating a trend for lower expression in SS. The consistency between qPCR and RNA-Seq is in agreement with previous studies that gene expression levels detected by these two methods are strongly correlated, although not completely consistent [29,30].

### Differentially expressed genes that are associated with host adaptive immunity

When the functions of all DE genes were analyzed, the top enriched GO (Gene Ontology) term was “immune system process” based on DAVID bioinformatics functional annotation. IPA functional analysis also showed that 31 down regulated DE genes were related to immune functions, and several of gene products were immune cell membrane receptors and cytokines (Table 4).

**Table 4:** Functions of 31 down-regulated DE genes in super-shedders that are associated with immune functions predicted by IPA.

Symbol	Entrez Gene Name	Location	Type (s)	Log 2 (fold change)*
***CD69***	*CD69 molecule*	Plasma Membrane	transmembrane receptor	-1.8
***CD180***	*CD180 molecule*	Plasma Membrane	other	-1.9
***TIMD4***	*T-cell immunoglobulin and mucin domain containing 4*	Plasma Membrane	other	-2.0
***RHOH***	*ras homolog family member H*	Plasma Membrane	enzyme	-2.0
***SIT1***	*signaling threshold regulating transmembrane adaptor 1*	Plasma Membrane	other	-2.3
***CD79A***	*CD79a molecule*, *immunoglobulin-associated alpha*	Plasma Membrane	transmembrane receptor	-2.4
***FAIM3***	*Fas apoptotic inhibitory molecule 3*	Plasma Membrane	other	-2.6
***CD19***	*CD19 molecule*	Plasma Membrane	transmembrane receptor	-2.6
***IL2RA***	*interleukin 2 receptor*, *alpha*	Plasma Membrane	transmembrane receptor	-2.7
***CD22***	*CD22 molecule*	Plasma Membrane	transmembrane receptor	-2.8
***MS4A1***	*membrane-spanning 4-domains*, *subfamily A*, *member 1*	Plasma Membrane	other	-2.9
***CD79B***	*CD79b molecule*, *immunoglobulin-associated beta*	Plasma Membrane	transmembrane receptor	-3.3
***CCR7***	*chemokine (C-C motif) receptor 7*	Plasma Membrane	G-protein coupled receptor	-3.6
***KLHL6***	*kelch-like family member 6*	Other	other	-2.0
***POU2AF1***	*POU class 2 associating factor 1*	Nucleus	transcription regulator	-2.0
***TCF7***	*transcription factor 7 (T-cell specific*, *HMG-box)*	Nucleus	transcription regulator	-2.0
***LEF1***	*lymphoid enhancer-binding factor 1*	Nucleus	transcription regulator	-3.1
***CCL19***	*chemokine (C-C motif) ligand 19*	Extracellular Space	cytokine	-2.2
***SPP1***	*secreted phosphoprotein 1*	Extracellular Space	cytokine	-2.9
***LTB***	*lymphotoxin beta (TNF superfamily*, *member 3)*	Extracellular Space	cytokine	-2.9
***CCL21***	*chemokine (C-C motif) ligand 21*	Extracellular Space	cytokine	-3.4
***CXCL13***	*chemokine (C-X-C motif) ligand 13*	Extracellular Space	cytokine	-4.3
***SASH3***	*SAM and SH3 domain containing 3*	Cytoplasm	other	-1.7
***GIMAP5***	*GTPase*, *IMAP family member 5*	Cytoplasm	other	-2.0
***FAM65B***	*family with sequence similarity 65*, *member B*	Cytoplasm	other	-2.1
***THEMIS***	*thymocyte selection associated*	Cytoplasm	other	-2.2
***SH2D1A***	*SH2 domain containing 1A*	Cytoplasm	other	-2.2
***PLA2G2A***	*phospholipase A2*, *group IIA (platelets*, *synovial fluid)*	Cytoplasm	enzyme	-3.6
***S100A12***	*S100 calcium binding protein A12*	Cytoplasm	other	-3.8
***S100A9***	*S100 calcium binding protein A9*	Cytoplasm	other	-5.0
***S100A8***	*S100 calcium binding protein A8*	Cytoplasm	other	-5.5

*log 2 (fold change) is log ratio of gene expression level in super-shedders to non-shedders.

**Fig 3:**
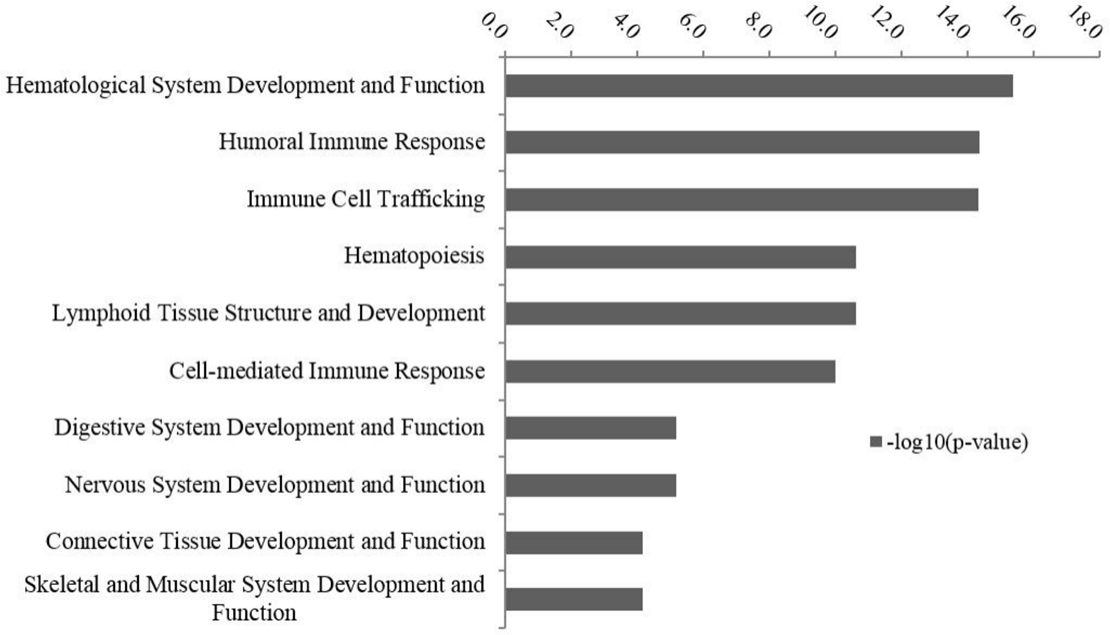
Top enriched functional categories by IPA for the differentially-expressed genes. The y-axis is on–log_10_ (P-value) scale: the greater the–log_10_ (P-value), the more likely that function is associated with the differentially expressed genes.

According to IPA functional analysis, the identified DE genes were involved in 56 physiological processes (IPA z-score ≤ -2.0 or ≥ 2.0) (Fig 3 and S4 Table). The “hypoplasia of lymphatic system” was the only function predicted to be increased in SS (S4 Table), and five down-regulated genes were involved in this function, including *Kelch-Like Family Member 6* (*KLHL6*), *POU2AF1*, *Ras Homolog Family Member H* (*RHOH*), *SAM And SH3 Domain Containing 3* (*SASH3*), and *Sirtuin 1* (*SIT1*) (Table 4). The transcription factor encoded by *POU2AF1* is involved in development of B-cells, and formation of germinal centers in peripheral lymphoid organs [31], while *RHOH* and *SITI* have been reported to be involved in regulation of maturation of T-cells [32,33]. Moreover, *SASH3* is involved in B- and T-cell proliferation [34], and *KLHL6* is suggested to play a role in B-cell maturation [35]. Down-regulation of these genes suggests possible disrupted maturation and proliferation of B- and T-cells in RAJ tissues.

Eleven down-regulated DE genes in SS were associated with the decreased functions in “quantity of lymphocytes”, “development of lymphocytes”, “activation of lymphocytes” and “lymphocytes migration”. These genes include *KLHL6*, *POU2AF1*, *SASH3*, *MS4A1*, *CD22*, *CD19*, *CD79a molecule*, *immunoglobulin-associated alpha (CD79A)*, *CD79b Molecule*, *Immunoglobulin-Associated Beta* (*CD79B)*, *CXCL13*, *CCL19*, and *CCL21* (Table 5). *KLHL6* is associated with B-cell activation and proliferation, and insufficient expression of this gene causes a reduction in B-cell subsets in mice [35]. The expression of *POU2AF1* is essential for B-cells activation and germinal center development [31], while *SASH3* is a signaling adapter gene expressed in lymphocytes, and decrease in its expression is associated with a decrease in activation and production of B-cells [34]. The *MS4A1* is important for B-cell proliferation, decrease in the number of B-cells was observed with insufficient expression of this gene [36]. The *CD22*, *CD79a*, *CD79b* and *CD19* encode B-cell specific membrane proteins, and down-regulation of these genes indicate a potential decrease in B-cell number [37,38]. The CD19 was also reported to promote the activation of B-cells, and its down-regulation may contribute to decreased activation of B-cells [39] in SS. The *CXCL13* gene encodes a chemoattractant of B-cells, and expression of this gene is required for migration of B-cells to gut associated lymphoid tissues and formation of lymphoid follicle [40]. The CCL19 and CCL21 are chemoattractants for B-cells, and CCL19 is associated with migration of B-cells from bone marrow to peripheral lymphoid tissues [40], while CCL21 regulates B-cells movement in secondary lymphoid tissues [41]. Down-regulation of these genes suggests possible reduction in B-cell quantity, development and migration in SS, which may lead to impairment in mucosal IgA production. As a result, the susceptibility to colonization by *E*. *coli* O157:H7 at RAJ of SS may be higher than NS.

**Table 5:** Decreased immune functions in super-shedders identified by the DE genes using IPA.

Associated immune cells	Functions Annotation	z-score	Associated molecules
	cell movement of T lymphocytes	-2.4	*CCL19*, *CCL21*, *CCR7*, *CD69*, *CXCL13*, *IL2RA*, *LTB*, *SPP1*
	T cell homeostasis	-2.4	*CD79A*, *IL2RA*, *LEF1*, *LTB*, *RHOH*, *SH2D1A*, *SPP1*, *TCF7*, *THEMIS*
**T-cells**	T cell development	-2.4	*CD79A*, *IL2RA*, *LEF1*, *LTB*, *RHOH*, *SH2D1A*, *SPP1*, *TCF7*, *THEMIS*
	quantity of T lymphocytes	-2.3	*CCL19*, *CCL21*, *CCR7*, *CD79A*, *IL2RA*, *LTB*, *POU2AF1*, *RHOH*, *SASH3*, *SH2D1A*, *SIT1*, *THEMIS*
	quantity of CD4+ T-lymphocytes	-2.2	*CD69*, *RHOH*, *SH2D1A*, *SIT1*, *THEMIS*
	quantity of regulatory T lymphocytes	-2.2	*CCL19*, *CCL21*, *IL2RA*, *LTB*, *THEMIS*
	quantity of lymphocytes	-2.6	*CCL19*, *CCL21*, *CCR7*, *CD19*, *CD22*, *CD79A*, *CD79B*, *CXCL13*, *IL2RA*, *KLHL6*, *LTB*, *MS4A1*, *POU2AF1*, *RHOH*, *SASH3*, *SH2D1A*, *SIT1*, *THEMIS*
	development of lymphocytes	-2.5	*CD19*, *CD79A*, *GIMAP5*, *IL2RA*, *LEF1*, *LTB*, *RHOH*, *SH2D1A*, *SPP1*, *TCF7*, *THEMIS*
	binding of lymphocytes	-2.2	*CCL19*, *CCL21*, *CD22*, *CXCL13*, *SH2D1A*
**T-, B-cell**	infiltration by lymphocytes	-2.2	*CCL19*, *CCL21*, *CCR7*, *IL2RA*, *LTB*
	activation of lymphocytes	-2.0	*CD19*, *CD69*, *PLA2G2A*, *POU2AF1*, *S100A9*, *SASH3*, *SH2D1A*, *SPP1*
	maturation of lymphocytes	-2.0	*CD19*, *LEF1*, *RHOH*, *TCF7*
	cell viability of lymphocytes	-2.4	*CD19*, *CD22*, *CD79A*, *GIMAP5*, *LEF1*, *TCF7*
	Lymphocyte migration	-2.1	*CCL19*, *CCL21*, *CCR7*, *CD69*, *CXCL13*, *IL2RA*, *LTB*, *SH2D1A*, *SPP1*
**Phagocyte, macrophage, dendritic, neutrophils**	cell movement of phagocytes	-2.7	*CCL19*, *CCL21*, *CCR7*, *CXCL13*, *LTB*, *S100A8*, *S100A9*, *SPP1*
**Neutrophils**	chemotaxis of neutrophils	-2.6	*CCL19*, *CCL21*, *CCR7*, *CD69*, *S100A8*, *S100A9*, *SPP1*
**Dendritic cells**	migration of dendritic cells	-2.2	*CCL19*, *CCL21*, *CCR7*, *CXCL13*, *SPP1*
NK cells*	activation of natural killer cells	-2.0	*CD69*, *S100A9*, *SH2D1A*, *SPP1*

* NK cells: natural killer cells.

The isolated lymphoid follicles (ILFs) in the bovine rectum were suggested to be the source of secretory antibodies in the gut, serving as the first line of defense in the gastrointestinal (GI) tract [42]. Lymphotoxin is necessary for the initiation of ILF development [43], and the down-regulation of *lymphotoxin beta* (*LTB*) indicate probable decrease in production of lymphotoxin (Table 4). Impaired ILF was reported to cause a 10-fold increase of segmented filamentous bacteria [44], and 100-fold increase of anaerobic bacteria in the small intestine of mice [45]. Others have observed that impairment in ILF function resulted in a 10 to 100-fold increase of *Enterobacteriaceae* colonization in the ileum of mice [46]. These observations suggest that ILFs play an important role in regulating the density of bacterial populations attached to the intestinal epithelium of the host. Down-regulation of *LTB* indicated a possibility that the RAJ of SS contains less well-developed ILF than NS, and thus we speculate that a higher diversity of microbes can be harbored by RAJ of SS. This hypothesis is supported by the observation of greater diversity of fecal microbiota in SS as compared to NS from the same animals [47], and a tissue colonized microbiota study is required to further confirm our speculation.

In addition to ILFs, functions on the “movement of T lymphocytes”, “quantity of T lymphocytes” and “T-cell development” decreased in the RAJ of SS as compared to NS (Table 5). This suggests that the cell-mediated immune response could also be related to super-shedding. T-cells are distributed among the epithelium (intraepithelial T-cells, IET) and the lamina propria, with IET providing rapid T-cell responses to numerous antigens and lamina propria T-cells undertaking regulatory functions [48]. The decreased function in the movement, development and quantity of T-cells may lead to the reduced accumulation of IET and lamina propria T-cells in SS, enabling *E*. *coli* O157:H7 biofilms to more readily form on the RAJ epithelium.

### Differentially expressed genes that are associated with host innate immunity

The DE analysis of genes also revealed a decrease in the functions of “cell movement of phagocytes”, “chemotaxis of neutrophils” and “migration of dendritic cells”, and “activation of natural killer cells” (Table 5), suggesting less active innate immune protection in SS than NS.

Intestinal phagocytes, especially macrophage, can rapidly eliminate bacteria without eliciting a strong inflammatory response [49]. In SS, down-regulation of six cytokine and cytokine receptor genes including *CCL13*, *CCL21*, *CXCL13*, *SPP1*, *LTB* and *CCR7* (Table 5) suggest a reduction in the function of the movement of phagocytes, including macrophage, neutrophils and dendritic cells [50–53]. Down-regulation of two S100 family genes, *S100 Calcium Binding Protein A8* (*S100A8*) and *S100 Calcium Binding Protein A9* (*S100A9*), involved in movement of neutrophils may also be an indication of decreased chemotaxis of neutrophils [54] in SS. Also, the function of “migration of dendritic cells” was predicted to be decreased in SS as a result of down-regulation of *CCL19*, *CCL21*, *CCR7*, *CXCL13* and *SPP1* (Table 5). All these genes encode cytokines/cytokines receptors which positively regulate chemotaxis of dendritic cells [50,55–57] that plays an important role in mucosal immune functions, such as antigen presenting and activation of B- and T-cells. The decreased function of migration of dendritic cells suggests reduced innate immune protection through fewer activated effector B- and T-cells distributed in the epithelium and lamina propria at the RAJ of SS. In addition, down-regulation of *CD69*, *S100A9*, *SH2D1A* and *SPP1* (Table 4) in SS is suggested to be associated with the decreased function in “activation of natural killer cells (NK cells)” (Table 5). The *CD69* gene encodes a cell membrane protein of activated NK cells [58] and the *S100A9*, *SH2D1A* and *SPP1* gene products have been reported to positively regulate activation of NK cells [59–61], and therefore, their down-regulation suggest a decreased NK cell activation at the RAJ of SS.

### Pathway enrichment analysis of differentially expressed genes

To further define the potential functional outcomes of the 58 DE genes, the KEGG_PATHWAY analysis was performed. Four pathways were enriched by DAVID Bioinformatics including the hematopoietic cell lineage (KEGG PATHWAY entry: bta04640), cytokine-cytokine receptor interaction (KEGG PATHWAY entry: bta04060), the chemokine signaling pathway (KEGG PATHWAY entry: bta04062), and the B-cell receptor signaling pathway (KEGG PATHWAY entry: bta04662). Four down-regulated DE genes including *IL2RA*, *CD19*, *CD22* and *MS4A1*were found to be associated with the hematopoietic cell lineage pathway in SS. The down-regulation of *IL2RA* suggests the decrease in T-cell differentiation, and the down-regulation of *CD19*, *CD22*, *IL2RA* and *MS4A1* suggests the decrease in B-cell differentiation. Six down-regulated genes involved in cytokine-cytokine receptor interaction and chemokine signaling pathways were also identified, including *CCL19*, *CCL21*, *CCR7*, *CXCL13*, *IL2RA* and *LTB*. The down-regulation of these genes may be associated with a reduction in the chemotaxis of leukocytes in SS. Four down-regulated genes, *CD19*, *CD22*, *CD79a* and *CD79b* involved in the B-cell receptor signaling pathway also indicate a potential reduction in antibody production at the RAJ of SS. The pathway analysis of DE genes further suggested the potentially reduced innate and adaptive immune functions at RAJ of SS as identified above in the functional analysis.

### miRNA profiling and differentially expressed miRNAs for bovine rectal tissue

**Fig 4:**
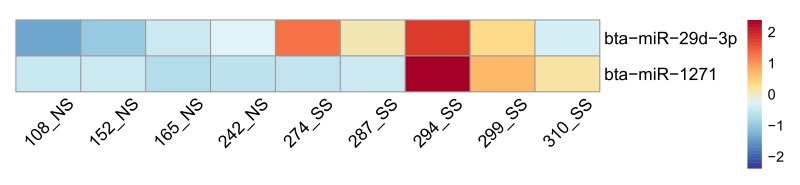
Differentially expressed miRNAs in recto-anal junction. The heatmap was created using pheatmap function in R package. Blue colors represent lower expression, and red color represent higher expression. Scaled reads per million (RPM) values were indicated by the color bar. NS, non-shedders; SS, super-shedders.

To identify the potential regulatory mechanism on gene expression associated with SS, we further studied miRNAs, a group of non-coding RNAs that post transcriptionally regulate gene expression (suppress target genes expression) [62]. In total, 33.5 M reads were generated with 399 ± 16 miRNA identified in each sample (S5 Table). Two miRNAs, bta-miR-1271 and bta-miR-29d-3p were found to be up-regulated in SS as compared to NS (FDR < 0.1, log 2 (fold change) > 1.0) (Fig 4). The bta-miR-29d-3p belongs to miR-29 family which plays a regulatory role in adaptive and innate immune responses [63], and up-regulation of this particular miRNA may suppress immune responses. Both target prediction and correlation analysis between gene and miRNA expression levels indicate that bta-miR-29d-3p potentially targets one of DE genes, *G-protein signaling 13* (*RGS13*). *RGS13* is expressed in lymphoid tissues and has been detected in B-cells [64], and down-regulation of *RGS13* could lead to impaired chemotaxis and maturation of B-cells in germinal center [65]. Up-regulation of bta-miR-29d-3p and down-regulation of *RGS13* in the SS (S6 Table) in SS, indicates that this miRNA-mRNA pair is possibly involved in the impaired migration and development of B-cells in SS. Further investigations are needed to identify the role of *RGS13* in *E*. *coli* O157:H7 shedding, and the regulatory mechanism of bta-miR-29d-3p.

### Other factors possibly contributing to decrease in host immune functions in SS

Environmental and bacterial factors could also contribute to the decrease in the immune function of SS. Since the cattle were from the same feedlot, it would be reasonable to assume that the environmental factors were similar for each of the sampled individuals. Colonization of the RAJ itself by *E*. *coli* O157:H7 could impair host immune responses as several EHEC secretive proteins, such as NleB (non-LEE-encoded type III effector B), NleC, NleE and NleH have been shown to impair host innate immunity by disrupting NF-κB pathways [7]. Furthermore, Shiga-toxins produced by EHEC could also suppress cellular immune responses, as shown by the down-regulation of membrane surface proteins on macrophages [66] and a reduction in the activation and proliferation of B- and T-cells [67]. Therefore, *E*. *coli* O157:H7 mediating mechanisms may also contribute to the observation that the rectal tissue of SS exhibited less effective innate and adaptive immune protection.

Based on above detected changes in rectal transcriptomes, it is difficult to conclude if the potentially reduced immune functions in SS are an inherent property of these animals, or those immune functions were repressed by *E*. *coli* O157 mediated activities. It is worthy to mention that although current study is the first to clearly present a connection between host gene expression and super-shedding phenomenon, there are limitations in terms of discrepancy of sampling time between tissue collection and feces *E*. *coli* O157 enumeration. To define whether such connection can be biologically meaningful and to gain fundamental understanding of super-shedding, a complicated biological phenomenon, as well as to investigate immunomodulatory of *E*. *coli* O157, further research including (1) synchronized tissue and fecal sampling from alive SS and (2) longitudinal monitoring both host gene expression and *E*. *coli* O157 shedding levels is required.

## Conclusion

Transcriptomic (mRNAs and miRNAs) analysis of rectal tissues from NS and SS suggested a potentially decreased function in the number and movement of phagocytes and lymphocytes in the rectum of SS. Because the ILFs, epithelium and lamina propria contain the majority of mucosal immune cells at the RAJ, our findings reflect potential impairment of these rectal lymphoid structures in SS. Based on the transcriptomic analysis, the rectal mucous membrane ILF of SS may contain less lymphocytes, macrophage and dendritic cells than NS. The less expression of immune related genes also indicates a failure to mount adequate immune protection in RAJ of SS which can result in enhanced colonization by *E*. *coli* O157:H7, and/or that colonization by the bacterium itself suppresses immune function. Our study showed contradicted results comparing to previous studies on *E*. *coli* O157:H7 experimentally challenged cattle that innate and adaptive immune responses could be promoted at the RAJ [68], including increased neutrophil infiltration and IgA production [68] and increased release of cytokine and proliferation of T-cells and NK cells [8]. Different from previous studies, we focused on cattle naturally-colonized with *E*. *coli* O157:H7, which may have different response mechanisms comparing to experimentally challenged condition. In addition, sampling from commercial feedlot limited our sampling approaches, rendering a necessity of further verification for current data. However, current findings not only justify further research, but also raised an important question: is the repressed immune protection a result of *E*. *coli* O157 activity or an inherent property of SS? Improved sampling technique that allow synchronized rectal tissue and feces sampling from SS would potentially answer this question. To advance our understanding on the mechanisms of *E*. *coli* O157:H7 colonization at the RAJ of cattle, and the mechanisms resulting in the super-shedding phenotype as well as to verify our findings, immunological and proteomic studies are also required. The transcriptomes of other regions of the bovine gut should also be investigated to define if there are any specific genes expressed at RAJ that make it the preferred site for colonization by *E*. *coli* O157:H7 in the gastrointestinal tract. From our results, the transcriptome of non-persistent super-shedders appears to differ from those of non-shedders, largely due to factors linked to immune function. This is an important preliminary step in helping to identify pre-disposing factors for super-shedding, although additional information is required to define the actual mammalian and bacterial cellular response that lead to super-shedding.

## Supporting Information

S1 Table Genes used for core transcriptomic analysis for RAJ tissue. (XLSX)

S2 Table Top physiological functions of rectal tissue and associated molecules. (XLSX)

S3 Table The Ensembl ID, Gene Symbol and log 2 (fold change) of DE genes. (XLSX)

S4 Table Functions significantly changed. Predicted by IPA, functions with z-sore < -2 or > 2 in super-shedders.
(XLSX)

S5 Table miRNAs identified in RAJ tissues of each animal. Expression level indicated by reads per million (RPM).
(XLSX)

S6 Table Targets of bta-miR29d-3p and bta-miR-1271; miRNA-target Pearson correlation coefficient and P-value. (XLSX)
